# Adiponectin Regulates the Polarization and Function of Microglia via PPAR-γ Signaling Under Amyloid β Toxicity

**DOI:** 10.3389/fncel.2017.00064

**Published:** 2017-03-07

**Authors:** Juhyun Song, Seong-Min Choi, Byeong C. Kim

**Affiliations:** ^1^Department of Biomedical Sciences, Center for Creative Biomedical Scientists at Chonnam National UniversityGwangju, South Korea; ^2^Department of Neurology, Chonnam National University Medical SchoolGwangju, South Korea

**Keywords:** adiponectin, Acrp30, microglia, inflammation, amyloid beta (Aβ), cytokines, peroxisome proliferator-activated receptor (PPAR)-γ

## Abstract

Alzheimer’s disease (AD), characterized by the abnormal accumulation of amyloid beta (Aβ), is gradually increasing globally. Given that AD is considered a neuroinflammatory disease, recent studies have focused on the cellular mechanisms in brain inflammatory conditions that underlie AD neuropathology. Microglia are macrophage cells in the central nervous system (CNS) that are activated in response to Aβ condition. The function of microglia contributes to the neuroinflammation in AD brain, suggesting that microglia regulate the production of inflammatory mediators and contribute to the regeneration of damaged tissues. Adiponectin, an adipokine derived from adipose tissue, has been known to regulate inflammation and control macrophages during oxidative stress conditions. In present study, we investigated whether adiponectin influences the polarization and function of microglia under Aβ toxicity by examining alterations of BV2 microglia function and polarization by Acrp30 (a globular form of adiponectin) treatment using reverse transcription PCR, western blotting and immunofluorescence staining. Acrp30 promoted the induction of the M2 phenotype, and regulated the inflammatory responses through peroxisome proliferator-activated receptor (PPAR)-γ signaling under Aβ toxicity. In addition, Acrp30 boosted the capacity of Aβ scavenging in microglia. Taken together, we suggest that adiponectin may control the function of microglia by promoting anti-inflammatory responses through PPAR- γ signaling. Hence, we conclude that adiponectin may act as a critical controller of microglia function in the AD brain.

## Introduction

Alzheimer’s disease (AD) is the most common cause of dementia, manifesting as memory decline and cognitive dysfunction (Bubu et al., 2016; De Strooper and Karran, 2016; Tarantini et al., 2016). AD is defined by two pathological characteristics: amyloid β-protein (Aβ) deposition in senile plaques and phosphorylated tau in neurofibrillary tangles (Goate, 2006). Excessive accumulation of Aβ has been shown to lead to the cognitive impairments in AD (Hardy and Selkoe, 2002; Hardy et al., 2014), and has been shown to trigger inflammation (Del Bo et al., 1995; Combs et al., 2001; Takata et al., 2003; Lindberg et al., 2005; Kempuraj et al., 2016; Stamouli and Politis, 2016) through the production of cytokines and other inflammatory mediators. Several studies of AD brains reported the presence of activated glial cells around the Aβ plaques (Mehlhorn et al., 2000; Rogers and Lue, 2001); (uroff, 2017 #365). Microglia, the resident macrophages of the central nervous system (CNS; Nakamichi et al., 2013; Ginhoux and Prinz, 2015), constitute 10%–15% of brain cells and play a critical role in CNS homeostasis (Solé-Domènech et al., 2016). Previous studies demonstrated that microglia polarizes into two states, M1 like phenotype and M2 like phenotypes (Francos-Quijorna et al., 2016; Lee et al., 2016). M1 like phenotype microglia is related with pro inflammatory response’s induction, whereas M2 like phenotype microglia is associated with neuroprotective roles (Eggen et al., 2013; Gertig and Hanisch, 2014; Natoli and Monticelli, 2014; Plastira et al., 2016; Xu et al., 2016). The polarization of microglia is important in CNS homeostasis as it has been shown to affect learning and memory (Tremblay et al., 2011, 2015; Parkhurst et al., 2013). Numerous studies have suggested that microglia are activated in the AD brain and contribute to the prevention of Aβ formation through phagocytosis of Aβ (Rogers et al., 2002; Doens and Fernández, 2014). Several studies have demonstrated that M2 type microglia reduce inflammation in the brain (Weekman et al., 2014; Latta et al., 2015) and ameliorate cognitive dysfunction during Aβ toxicity (Zhu et al., 2016). These data highlight the necessity to elucidate the mechanisms underlying Aβ clearance by microglia, thereby attenuating AD progression. Adiponectin is encoded by the *ADIPOQ* gene (Maeda et al., 1996) and exerts multiple effects by binding to two specific receptors, AdipoR1 and AdipoR2 (Yamauchi et al., 2003; Hug et al., 2004). Adiponectin receptors exist in various organs, including the liver and brain (Kim et al., 2013; Kaminski et al., 2014). Previous researches have demonstrated that adiponectin acts as an insulin sensitizer, an anti-inflammatory regulator and an anti-atherosclerotic molecule (Kadowaki et al., 2006; Renaldi et al., 2009). Recent research has focused on the effect of adiponectin on macrophages (Elfeky et al., 2016; Masamoto et al., 2016; Wang et al., 2016); specifically, that adiponectin may not only act as a key regulator of inflammatory response in metabolic diseases, but also in CNS diseases (Wang et al., 2016). In the present study, we investigate whether adiponectin contributes to the polarization and the scavenger capacity of microglia under Aβ toxicity. The results provide significant evidence for the therapeutic potential of adiponectin to mitigate AD pathology by regulating the function of microglia.

## Materials and Methods

### Animal Experiments

5xFAD male transgenic mice (5 months of age) with detectable Aβ_42_ production in the brain were purchased from The Jackson Laboratory (Bar Harbor, ME). Wild-type male C57BL/6 mice (25 g, 5 months of age) were provided by Koatech (Koatech, Pyeongtaek, South Korea). In the present study, we used four mice in each groups. This study was carried out in accordance with the recommendations of “96 Guidance for Animal Experiments”, established by the “Animal Ethics Committee” at Chonnam National University. The protocol was approved by the “Animal Ethics Committee” at Chonnam National University, approved study number 2016-84. The animals were housed in an air-conditioned room at 25°C with a 12 h dark/light cycle. All animals received human care with unlimited access to chow and water.

### Cell Culture and Drug Treatment

Murine BV2 microglial cells were cultured in Dulbecco Modified Eagle Medium (DMEM; Gibco, Grand Island, NY, USA) supplemented with 10% fetal bovine serum (Gibco), 100 μg/ml penicillin (Gibco) and 100 μg/ml streptomycin (Gibco). BV2 cells were grown in a humidified incubator at 37°C with 5% CO_2_. The cells were pretreated with Acrp30 (5 μg/ml; Sigma-Aldrich, St. Louis, MO, USA) for 24 h and then treated with Aβ (10 μM) for 24 h. GW9662 was used as the antagonist for PPAR-γ (Chen et al., 2016). BV2 cells were pretreated with GW9662 (10 μM) for 3 h.

### Aβ Oligomer Preparation

Oligomeric Aβ was prepared as previously described (An et al., 2013). One milligram of synthetic Aβ 42 peptide (American Peptide, Sunnyvale, CA, USA) was dissolved into 1 ml HFIP (1,1,1,3,3,3-hexafluoro-2-propanol). The solution should then be dried under a nitrogen stream. Re-dissolved the remaining film in 100% HFIP to a concentration of 1 mg/ml. Sonicated in bath sonicator for 5 min and dried under a nitrogen stream. Repeated the HFIP treatment twice. Then re-dissolved in 1 ml HFIP and dried for 2 h. After that the film was resuspended in 200 ul DMSO to obtain a 1 mM Aβ stock solution. For 10 μM Aβ 42 treatment, 10 ul of Aβ 42 1 mM was dilute into 10 ml DMEM cell culture media and incubated for 12 h at 37°C.

### Preparation of the AdipoR1 and AdipoR2 Targeting siRNA

The siRNA for AdipoR1 and AdipoR2 (5 μM) was prepared to silence AdipoR1 siRNA sc-60123 (Santa Cruz Biotechnology, Santa Cruz, CA, USA) and AdipoR2 siRNA sc-46756 (Santa Cruz Biotechnology). For the transfection of siRNA, a 5 μM final concentration of siRNA AdipoR1 and siRNA AdipoR2 were combined with lipofectamine 2000 (Invitrogen, Carlsbad, CA, USA) in Opti-MEM medium, and incubated at room temperature for 20 min. The mixture was added to the BV2 cells in six well plates. After 2 days, the cells were collected for total protein or RNA extraction.

### Determination of Nitrite

BV2 microglia were seeded onto 96-well plates and pre-treated with Acrp30 (5 μg/ml) for 24 h prior to stimulation with 10 μM of Aβ. Supernatants in all groups were measured by nitric oxide (NO) production using Griess reagent (Sigma-Aldrich). The reagents (100 μl) were added to the plate and incubated for 30 min. The absorbance of supernatants was measured at 540 nm using an ELISA reader (Bio-Rad, Irvine, CA, USA).

### ELISA Assay

BV2 cells were plated in 6-well plates (5 × 10^5^ cells/ml) and incubated with Acrp30 (5 μg/ml) in the presence of Aβ 10 μM for 24 h. The production of TNF-α was checked by Cymax Mouse TNF-α ELISA kit (AbFrontier, Seoul, Korea) per manufacturer’s instructions. The absorbance at 450 nm was assessed by ELISA (Bio-Rad).

### Phagocytosis Assay

The phagocytosis assay was conducted using the Cayman chemical phagocytosis kit (Cayman Chemical, Ann Arbor, MI, USA). To check opsonization of target particles, all phagocytosis assays were conducted in complete DMEM media. Additionally, in some experiments, labeled particles were preopsonized by incubation at 37°C for 3 h with 5 mg/mL immunoglobulin G (IgG; Beletskii et al., 2005; Cayman Chemical).

### Western Blot Analysis

The BV2 cells were washed with phosphate-buffered saline (PBS) and collected. Cell pellets were lysed with ice-cold RIPA buffer (Sigma-Aldrich). The lysates were centrifuged at 15,900 g for 30 min at 4°C to produce whole-cell extracts. Protein (30 μg) in cells was separated on a 10% SDS-polyacrylamide gel and transferred onto a polyvinylidene difluoride membrane. After blocking with 5% skim milk prepared in Tris-buffered saline-Tween (20 nM Tris [pH 7.2], 150 mM NaCl, 0.1% Tween 20) for 1 h, 30 min at room temperature, immunoblots were incubated for 18 h at 4°C with primary antibodies that detect AdipoR1 (1:1000, Abcam, Cambridge, MA, USA), AdipoR2 (1:1000, Abcam, Cambridge, MA, USA), CD86 (1:1000, Cell Signaling, Danvers, MA, USA), p-NF-κB (1:1000, Cell Signaling), PPAR-γ (1:1000, Cell Signaling, Danvers, MA), p-PPAR-γ (1:1000, Santa Cruz Biotechnology, Santa Cruz, CA, USA) and β-actin (1:1000; Millipore, Billerica, MA, USA). All blots were then incubated with appropriate secondary antibodies (Abcam, Cambridge, MA, USA) for 1 h 30 min at room temperature. All blots were visualized using ECL solution (Millipore, Billerica, MA, USA).

### Quantitative Real-Time PCR

To examine the amount of CD86, CD206, iNOS and TNF-α mRNA in BV2 cells, quantitative real-time PCR was performed using each primer. Cellular RNA was extracted from BV2 cells using Trizol reagent (Invitrogen). RNA was mixed with One Step SYBR^®^ Prime Script TM RT-PCR Kit II (Takara, Otsu, Shiga, Japan) and specific primers in a total reaction volume of 50 μl. PCR was performed using the following each primers (5′ to 3′): CD86 (F): GTA TTT TGG CAG GAC CAG GA, (R): GCC GCT TCT TCT TCT TCC AT, CD206 (F): GAG GGA AGC GAG AGA TTA TGG A, (R): GCC TGA TGC CAG GTT AAA GCA, iNOS (F): GGG AAT CTT GGA GCG AGT TG, (R): GTG AGG GCT TGG CTG AGT GA, TNF-α (F): CGT CAG CCG ATT TGC TAT CT, (R): CGG ACT CCG CAA AGT CTA AG, GAPDH (F): GAC AAG CTT CCC GTT CTC AG, (R): GAG TCA ACG GAT TTG GTC GT. Amplification cycles were carried out at 52°C for 5 min, 95°C for 10 s, 95°C for 5 s, 58°C for 35 s and 65°C for 15 s. Quantitative SYBR Green real time PCR was performed with Takara PCR System (Takara, Otsu, Shiga, Japan). GAPDH was used as an internal control. The ΔCt values of treated cells were compared to those of normal, control cells (Popivanova et al., 2008).

### Reverse Transcription PCR

Total RNA in BV2 cells was isolated using Trizol Reagent (Gibco). RT-PCR reaction was conducted by using the Invitrogen One step III^TM^ Reverse Transcription PCR kit (Invitrogen). cDNA synthesis from mRNA and sample normalization were conducted. PCR was performed using the following thermal cycling conditions: 95°C for 10 min;, 40 cycles of denaturing at 95°C for 15 s, annealing at 60°C for 30 s, elongation at 72°C for 30 s, final extension at 72°C for 5 min and holding at 4°C. PCR was performed using the following primers (5′ to 3′): AdipoR1 (F): CCCACCATGCACTTTACTAT, (R) CACCATAGAAGTGGACGAAA, AdipoR2 (F): CAACCTTGCTTCATCTACCT, (R): CTAGCCATAAGCATTAGCCA, CD36 (F): GAG CCA TCT TTG AGC CTT CA, (R): TCA GAT CCG AAC ACA GCG TA, PPAR- γ (F): CAA TCC GAA TTT TTC AAG GGT GCC A, (R): GAG CAC CTT GGC GAA CAG CTG AGA G, iNOS (F): GGG AAT CTT GGA GCG AGT TG, (R): GTG AGG GCT TGG CTG AGT GA, TNF-α (F): CGT CAG CCG ATT TGC TAT CT, (R): CGG ACT CCG CAA AGT CTA AG, GAPDH (F): GAC AAG CTT CCC GTT CTC AG, (R): GAG TCA ACG GAT TTG GTC GT. PCR products were electrophoresed in 1% agarose gels. All samples were normalized with GAPDH.

### Immunocytochemistry

BV2 cells were washed with PBS and permeabilized for 20 min with 4% paraformaldehyde (Sigma-Aldrich). The cells were incubated with the primary antibodies overnight at 4°C. The primary antibody anti-rabbit CD86 (1:500, Cell Signaling) as a marker of M1 phenotype microglia (Gong et al., 2016) was used. After incubation, BV2 cells were washed twice with PBS and incubated with specific secondary antibody for 1 h 30 min at room temperature. The cells were counterstained with 1 μg/ml 4′,6-diamidino-2-phenylindole (DAPI, 1:100, Invitrogen) for 10 min at room temperature. Images were obtained using an LSM 520 confocal microscope (Carl Zeiss, Thornwood, NY, USA).

### Immunohistochemistry

Brain sections (20 μm) were mounted onto coated glass slides (Thermo Scientific, Waltham, MA, USA), and fixed in cold acetone for 10 min at −20°C. The slides were washed in Tris-buffered saline and incubated with 0.3% H_2_O_2_ in methanol. To block nonspecific labeling, sections were incubated in 5% bovine serum albumin (Sigma-Aldrich) and diluted in PBS for 80 min before the addition of primary and secondary antibody. Primary antibodies for anti-rabbit CD86 (1:100, Cell Signaling) and anti-rabbit Iba-1 (1:100, Cell Signaling) were applied to the samples overnight at 4°C, followed by 1 h 30 min incubation with appropriate florescence secondary antibody (1:100, Millipore), and three washes in PBS for 5 min each. After three washes in 0.1% PBS with Tween-20 (PBST), the sections were incubated with secondary antibody for 1 h 30 min in the dark at room temperature. After three washes in PBS, all sections were incubated with 1 μg/ml DAPI (Sigma-Aldrich, St. Louis, MO, USA) and visualized under a LSM 520 confocal microscope (Carl Zeiss).

### Statistical Analysis

The data were analyzed by SPSS 18.0 software (IBM Corp., Armonk, NY, USA). All results are expressed as mean ± standard deviation (SD). Statistical significance was determined using one-way analysis of variance (ANOVA) followed by Turkey’s multiple comparison and Student’s *t*-test. Differences were considered statistically significant at *p* < 0.05.

## Results

### The Activation and Polarization of Microglia in AD Mouse Brain

**Figure 1:**
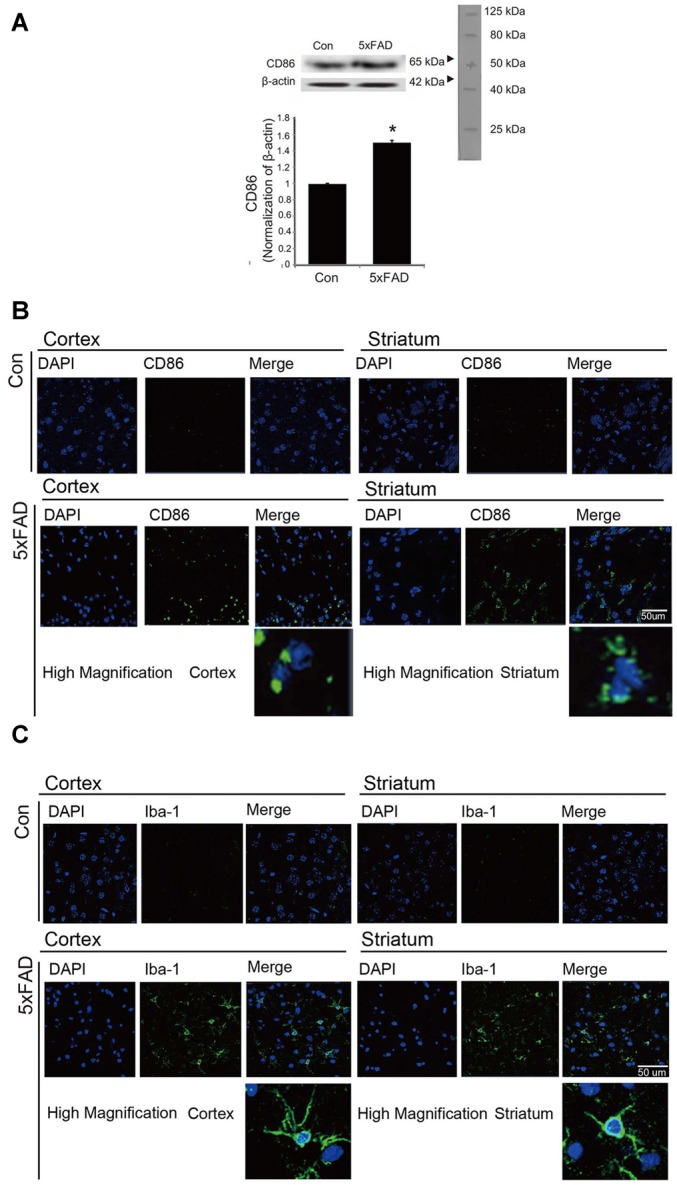
**The expression of CD86 and Iba-1 in 5xFAD mouse brain. (A)** Western blotting band showed increased CD86 protein level in the 5xFAD mouse brain. Data are expressed as mean ± SEM. Each experiment was conducted three times per condition. β-actin served as a control. Differences were considered statistically significant at *p < 0.05 (compared to control). Immunofluorescence images showed increased expression of CD86 **(B)** and Iba-1 **(C)** in the cortex and striatum of the 5xFAD mouse brain. Scale bar: 50 μm, Con: normal mouse, 5xFAD: 5xFAD mouse, CD86: green, Iba-1: green, 4′,6-diamidino-2-phenylindole (DAPI): blue.

**Figure 2:**
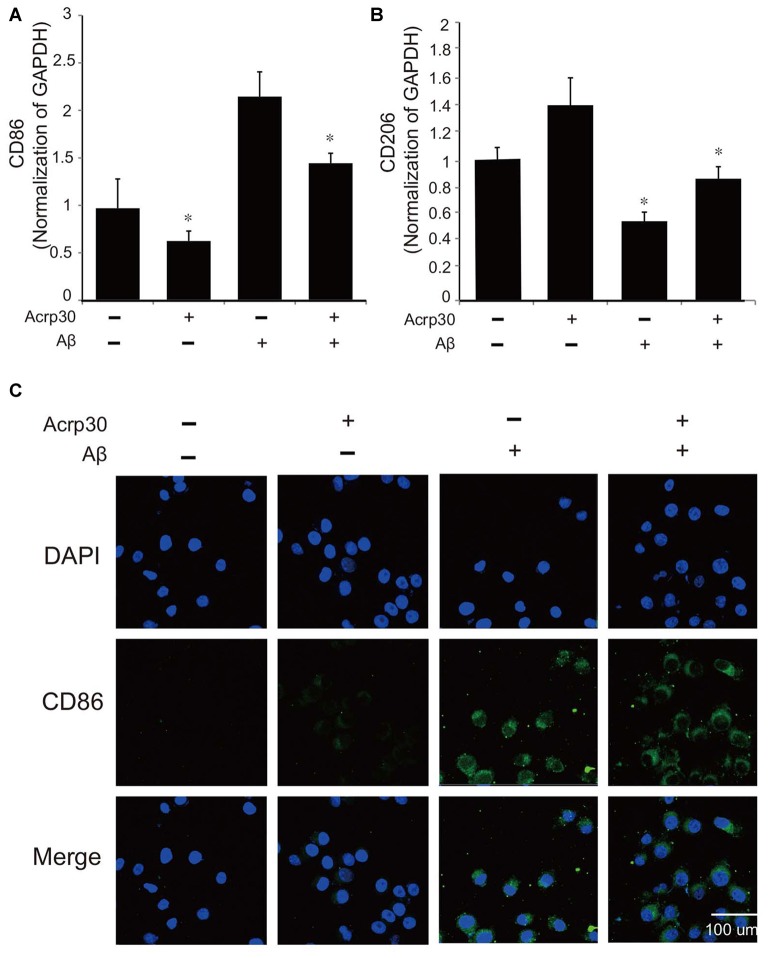
**The expression of CD86 and CD206 in amyloid beta (Aβ)-treated BV2 microglia.** The mRNA level of CD86 **(A)** and CD206 **(B)** was detected by RT PCR. Data are expressed as mean ± SEM. Each experiment was conducted three times per condition. GAPDH served as a control. Differences were considered statistically significant at *p < 0.05 (compared to control). **(C)** Immunofluorescence images showed the reduction of CD86 by Acrp30 pretreatment in Aβ- exposed BV2 microglia. Acrp30: Acrp30 5 μg/ml pretreatment, Aβ: Aβ 10 μM treatment for 24 h, Scale bar: 100 μm, CD86: green, 4′,6-diamidino-2-phenylindole (DAPI): blue.

To confirm the activation of microglia in the mouse brain, we detected the protein level of CD86 as a marker of activated microglia using western blot (Figure 1A), and the expression of CD86 and Iba-1 using immunohistochemistry (Figures 1B,C). Our data showed higher activation of microglia (increased CD86 protein level and a greater percentage of CD86 and Iba-1 positive cells) in the AD compared to the control mouse brain (Figures 1A–C). These results suggest that microglia are activated in an AD brain with Aβ accumulation. To assess the polarization of microglia with Aβ treatment and Acrp30 (a globular form of adiponectin) pretreatment, we assessed the mRNA level of CD86 as a marker of M1 phenotype microglia and of CD206 as a marker of M2 phenotype microglia (Figure 2). The mRNA level of CD206 was reduced while the mRNA level of CD86 was markedly increased in Aβ-exposed microglia (Figures 2A,B). Acrp30 reduced the expression of CD86 and increased the expression of CD206 in BV2 microglia (Figures 2A,B). Furthermore, Acrp30 pretreatment reduced CD86 mRNA level and increased CD206 mRNA level in spite of Aβ treatment (Figures 2A,B). Immunostaining data showed a decrease in CD86 expression in Aβ-exposed microglia with Acrp30 pretreatment (Figure 2C).

### The Activation and Polarization of Microglia in AD Mouse Brain

**Figure 3:**
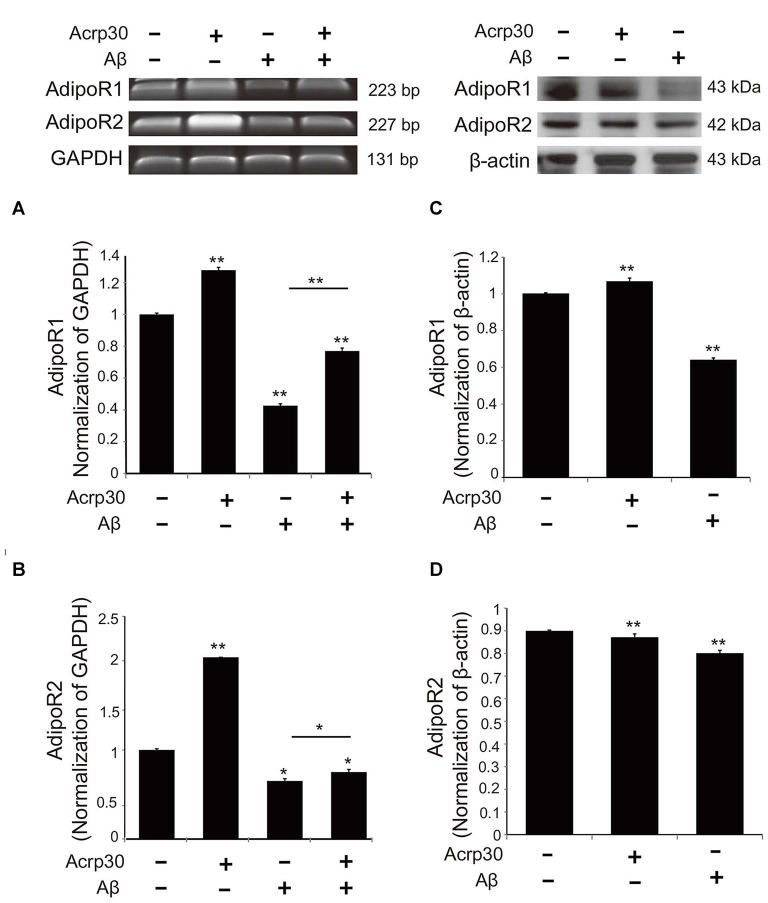
**The expression of adiponectin receptors in BV2 microglia.** The mRNA levels of AdipoR1 **(A)** and AdipoR2 **(B)** were measured by RT-PCR. The protein levels of AdipoR1 **(C)** and AdipoR2 **(D)** were detected by western blotting analysis. Data are expressed as mean ± SEM. Each experiment was conducted three times per condition. β- actin served as a control. Differences were considered statistically significant at *p < 0.05, **p < 0.001 (compared to control). Acrp30: Acrp30 5 μg/ml pretreatment, Aβ: Aβ 10 μM treatment for 24 h.

To examine the changes of adiponectin receptors in microglia under Aβ toxicity, we conducted reverse transcriptional PCR (Figures 3A,B) and western blotting (Figures 3C,D). The mRNA levels (Figures 3A,B) and protein levels (Figures 3C,D) of AdipoR1 and AdipoR2 were reduced in Aβ-treated microglia. Acrp30 promoted the expression of AdipoR1, whereas Acrp30 did not meaningfully change the expression of AdipoR2, in Aβ-treated microglia (Figures 3A,B). Notably, the protein level of AdipoR1 in microglia was decreased by Aβ treatment (Figure 3C).

### Acrp30 Treatment Leads to the Reduced Expression of Pro-Inflammatory Mediators in Aβ-Exposed Microglia

**Figure 4:**
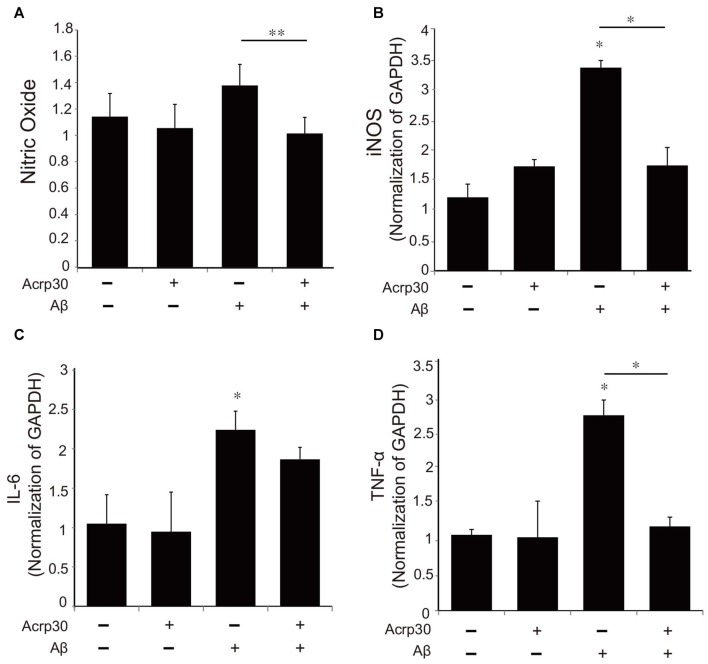
**The expression of pro-inflammatory mediators in Aβ-treated BV2 microglia. (A)** The production of nitric oxide (NO) was assessed by Griess reagent assay. Acrp30 reduced the production of NO in Aβ-treated microglia. The increased mRNA level of iNOS **(B)** and IL-6 **(C)** and TNF-α **(D)** were measured by RT PCR. Data are expressed as mean ± SEM. Each experiment was conducted four times per condition. GAPDH served as a control. Differences were considered statistically significant at *p < 0.05. **p < 0.001 (compared to control). Acrp30: Acrp30 5 μg/ml pretreatment, Aβ: Aβ 10 μM treatment for 24 h.

To examine alterations in the expression of pro-inflammatory mediators by Acrp30, we assessed the production of NO using the Griess assay (Figure 4A) and the mRNA of iNOS (Figure 4B). Moreover, we detected the mRNA level of IL-6 and TNF-α as pro-inflammatory cytokines using real time PCR in microglia (Figures 4C,D). Acrp30 treatment did not change the level of NO, iNOS, IL-6, or TNF-α in BV2 microglia, whereas the expression of these pro-inflammatory mediators were increased in Aβ-treated microglia. Acrp30 pretreatment led to a decrease in NO, iNOS, IL-6 and TNF-α in Aβ-treated microglia (Figures 4A–D).

### Acrp30 Reduced the Expression of TNF-α and iNOS in Aβ-Exposed Microglia

**Figure 5:**
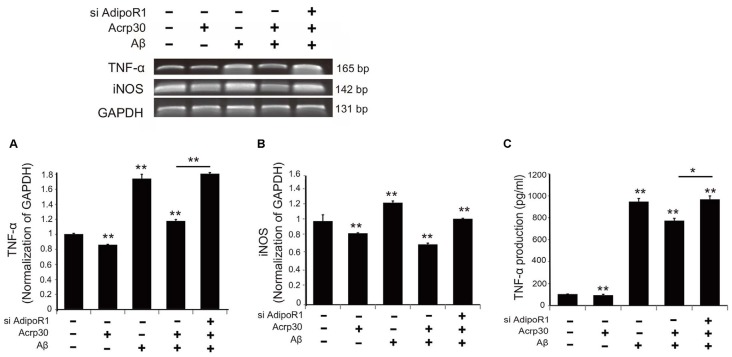
**The expression of pro-inflammatory mediators by suppressing AdipoR1 in Aβ-treated BV2 microglia.** The reduced mRNA level of TNF-α **(A)** and iNOS **(B)** and TNF-α **(C)** in Aβ-treated microglia were assessed by RT PCR. **(C)** The reduced production of TNF-α by Acrp30 in Aβ-treated microglia was increased by blocking AdipoR1. Data are expressed as mean ± SEM. Each experiment was conducted four times per condition. GAPDH served as a control. Differences were considered statistically significant at *p < 0.05. **p < 0.001 (compared to control). Acrp30: Acrp30 5 μg/ml pretreatment, Aβ: Aβ 10 μM treatment for 24 h.

To test for the expression of TNF-α and iNOS in Aβ-exposed microglia, we detected mRNA levels using reverse transcription PCR (Figure 5). The mRNA level of TNF-α in microglia under Aβ toxicity was significantly increased, while the mRNA level of TNF-α in Aβ-treated microglia was considerably reduced by Acrp30 pretreatment (Figure 5A). When we inhibited the expression of AdipoR1 using siRNA AdipoR1, we observed an increase in TNF-α mRNA level in Aβ-exposed microglia in spite of Acrp30 pretreatment (Figure 5A). In addition, the mRNA levels of iNOS and TNF-α showed the same expression patterns (Figure 5B), and the secretion of TNF-α and TNF-α mRNA level demonstrated similar patterns (Figure 5C). The knockdown of AdipoR1 did not reduce the expression of pro-inflammatory mediators (Figure 5).

### Acrp30 Increases the Ability of Phagocytosis in Aβ Exposed Microglia

**Figure 6:**
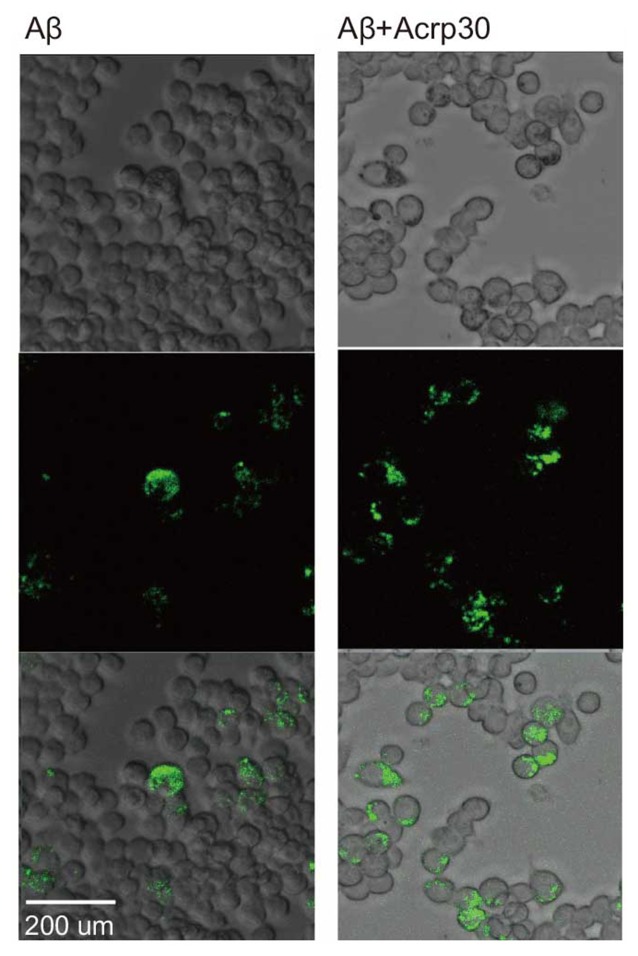
**The measurement of phagocytosis ability in Aβ-treated BV2 microglia.** Images showing the ability of phagocytosis in BV2 microglia. The green color shows the beads for determining the ability of phagocytosis in Aβ-treated microglia. Scale bar: 200 μm, Bead particles: green.

To examine the phagocytotic ability of microglia, we conducted the phagocytosis assay (Figure 6). Aβ-exposed microglia showed that the green beads were phagocytosed in many microglia. Acrp30 were increased the phagocytosis ability of microglia in Aβ toxicity (Figure 6). Thus, adiponectin promoted the phagocytosis ability of microglia in Aβ toxicity.

### Acrp30 Controls PPAR-γ Signaling in Aβ Exposed Microglia

**Figure 7:**
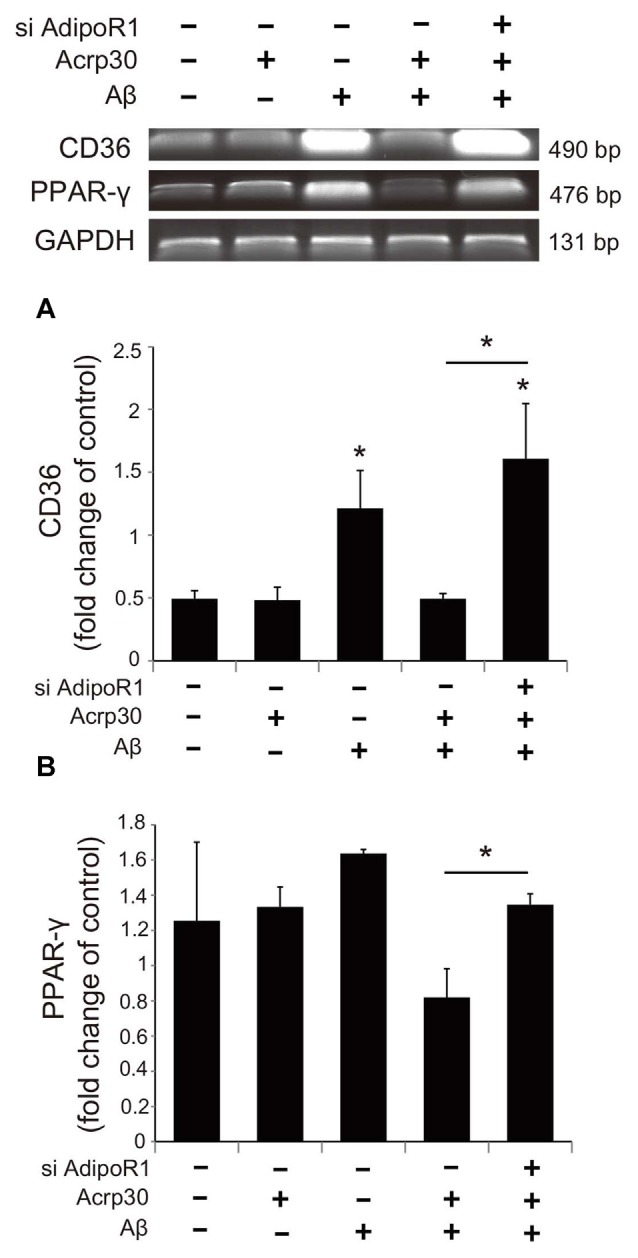
**The expression of CD36 and PPAR-γ in Aβ treated BV2 microglia.** The mRNA levels of CD36 **(A)** and PPAR-γ **(B)** were checked by RT PCR. The reduced mRNA levels of CD36 and PPAR-γ by Acrp30 in Aβ treated microglia were increased by inhibiting AdipoR1. Data are expressed as mean ± SEM. Each experiment was conducted four times per condition. GAPDH served as a control. Differences were considered statistically significant at *p < 0.05 (compared to control). Acrp30: Acrp30 5 μg/ml pretreatment, Aβ: Aβ 10 μM treatment for 24 h, siAdipoR1: siRNA AdipoR1 transfection.

**Figure 8:**
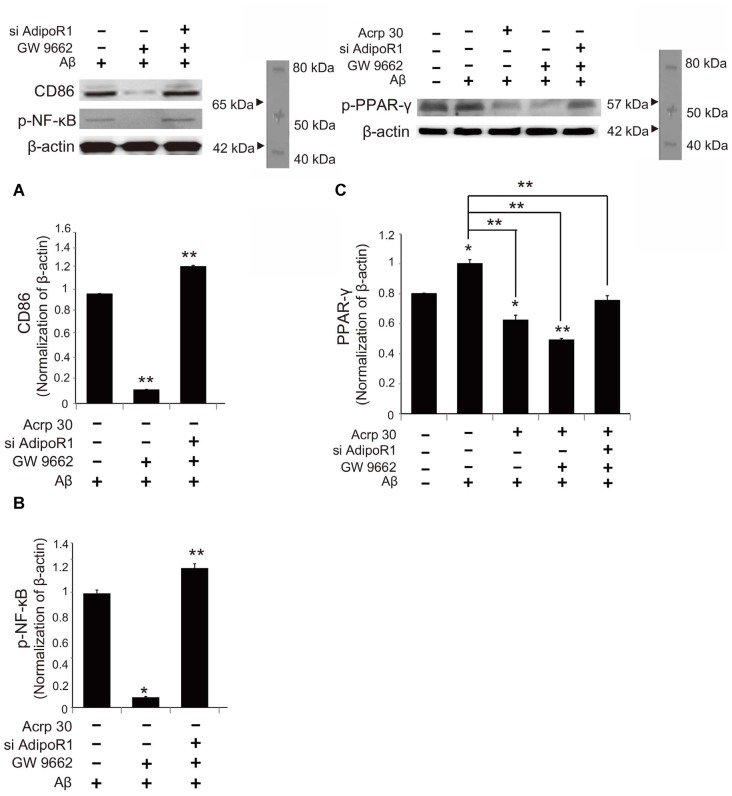
**The change in PPAR-γ signaling by inhibiting AdipoR1 in Aβ-treated BV2 microglia.** The protein levels of CD86 **(A)** and p-NF-κB **(B)** p-PPAR-γ **(C)** protein were detected by western blotting analysis. **(C)** The decreased p-PPAR-γ protein level by Acrp30 and GW9662 in Aβ-treated microglia was not changed by suppressing AdipoR1. Data are expressed as mean ± SEM. Each experiment was conducted four times per condition. GAPDH served as a control. Differences were considered statistically significant at *p < 0.05 (compared to control). Acrp30: Acrp30 5 μg/ml pretreatment, Aβ: Aβ 10 μM treatment for 24 h, siAdipoR1: siRNA AdipoR1 transfection, GW9662: PPAR-γ antagonist 10 μM. **p < 0.001.

To examine whether Acrp30 influences the expression of PPAR-γ in Aβ-treated microglia, we conducted real time PCR (Figure 7). The mRNA level of CD36 (a scavenger receptor for phagocytosis) was markedly increased in Aβ-treated microglia (Figure 7A). Acrp30 attenuated the expression of CD36 in microglia under Aβ toxicity (Figure 7A). The mRNA level of PPAR-γ was increased in Aβ treated microglia, while Acrp30 reduced the expression of PPAR-γ in microglia under Aβ toxicity (Figure 7B). To test whether Acrp30 directly regulates the PPAR-γ signaling in Aβ treated microglia, we examined CD86, p- NF-κB and PPAR-γ protein levels using western blotting analysis (Figure 8) and tested the effect of the PPAR-γ antagonist GW9662 in Aβ-treated microglia (Figure 8). Our results showed that GW9662 reduces the protein level of CD86 in spite of Aβ-treatment (Figure 8A). Reduced CD86 protein level in Aβ treated microglia with GW9662 was increased by inhibiting AdipoR1 (Figure 8A). The blocking of AdipoR1 reduced the function of PPAR-γ (decreased the expression of CD86) in Aβ-treated microglia (Figure 8A). In addition, GW9662 reduced the phosphorylation of NF-κB in spite of Aβ-treatment (Figure 8B). Reduced protein level of p-NF-κB in Aβ-treated microglia with GW9662 was increased by inhibiting AdipoR1 (Figure 8B). The protein level of PPAR-γ in Aβ-treated microglia was reduced by Acrp30 and GW 9662, whereas the protein level of PPAR-γ in Aβ-treated microglia with GW9662 was not changed by inhibition of AdipoR1 (Figure 8C). Based on our results, Acrp30 attenuated PPAR-γ signaling in microglia by mediating AdipoR1.

## Discussion

AD is characterized by the abnormal accumulation of Aβ and chronic neuroinflammation (Ramanathan et al., 2015; Scheltens et al., 2016). Clearance of Aβ and chronic inflammation are related to the polarization of and phagocytotic ability of microglia (Ide et al., 1996; Heese et al., 1998; Hutchinson et al., 2009; Tang and Le, 2016). The M2 phenotype microglia have been demonstrated to have a neuroprotective function in AD (Zhu et al., 2016). A recent study showed that M2 macrophage transplantation reduces neuroinflammation in the brain, promotes clearance of Aβ, and improves cognitive impairment (Zhu et al., 2016). Aβ and tau oligomers activate M1 pro-inflammatory responses and induce irreversible neuron loss (Tang and Le, 2016). The M1 phenotype microglia secrete pro-inflammatory mediators including TNF-α, IL-6 and NO, whereas anti-inflammatory M2 phenotype microglia produce anti-inflammatory mediators such as IL-10 and IL-4 under oxidative stress conditions (Tang and Le, 2016). In this study, we examined the alteration of cytokine expression by Acrp30 in Aβ-treated microglia. We found that adiponectin attenuates the expression of pro-inflammatory cytokines, and AdipoR1 may mediate the function of adiponectin secretion of inflammatory cytokines, considering that the expression of pro-inflammatory cytokines was not reduced in Aβ-treated microglia despite Acrp30 pretreatment.

In the present study, we found activated microglia *in vivo* in the AD mouse brain and *in vitro* under Aβ–induced inflammatory conditions. Increased expression of CD86, a known M1 receptor marker (Gong et al., 2016) was found in the cortex and striatum of 5xFAD mice, and in Aβ-treated BV2 microglia. Based on our results, we suggest that adiponectin might promote the polarization of M2 type microglia under Aβ toxicity conditions. Moreover, our results showed that adiponectin activates M2 phenotype microglia (Lovren et al., 2010). Increased CD206 and reduced M1 polarization was demonstrated by the reduction of pro-inflammatory cytokines such as TNF-α and IL-6, and the reduction of CD86 and iNOS expression.

Adiponectin regulates many processes by binding with specific receptors, AdipoR1 and AdipoR2 (Hug et al., 2004) and exists in various organs, including the brain (Kaminski et al., 2014). We observed more expression of AdipoR1 than AdipoR2 in BV2 microglia under Aβ induced toxicity. Previous studies demonstrated that downregulation of AdipoR1 attenuates anti-inflammatory responses (Zhang et al., 2016) and reduces the adiponectin’s beneficial actions in macrophages (Luo et al., 2013). We observed that the downregulation of AdipoR1 by siRNA AdipoR1 increases pro-inflammatory cytokines including TNF-α and iNOS production under Aβ toxicity in spite of Acrp30 pretreatment. Judging from our results, we assume that the inhibitory effect of adiponectin in pro-inflammatory cytokine production may be mediated by AdipoR1.

Fibrillar Aβ has been reported to interact with the cell surface receptor and leads to intracellular signaling, and then stimulates phagocytosis (D’Andrea et al., 2004; Mohamed and Posse de Chaves, 2011). CD36, one of the class B scavenger receptors, has been shown to be expressed in various cell types and bind with diverse ligands (Febbraio et al., 2001), and to participate in the internalization of numerous small particles, including Aβ (Koenigsknecht and Landreth, 2004) and apoptotic cells (Fadok et al., 1998). CD36 has been shown to uptake Aβ (Moore et al., 2002), and is involved in the production of pro-inflammatory mediators (Janabi et al., 2000; Stuart et al., 2005; Baranova et al., 2008). Several studies have demonstrated that CD36 boosts pro-inflammatory responses (Janabi et al., 2000; Moore et al., 2002; Okamura et al., 2009; Silverstein and Febbraio, 2009) and phagocytosis (Erdman et al., 2009). Studies have also shown that the activation of macrophages promotes the expression of CD36 by generation of ligands of PPAR-γ (Huang et al., 1999; Berry et al., 2007) and adiponectin inhibits the expression of scavenger receptor CD36 (Ouchi et al., 2001). Based on the current results, we hypothesize that adiponectin can attenuate the expression of CD36, leading to the production of pro-inflammatory cytokines.

Moreover, previous studies have demonstrated that PPAR-γ can mediate the conversion of the microglia phenotype (Yamanaka et al., 2012; Saijo et al., 2013). Our results indicate that PPAR-γ may induce M2 phenotype microglia under Aβ toxicity via adiponectin treatment. Several studies have demonstrated that PPAR-γ agonists alleviate neuroinflammation in AD models by acting as anti-inflammatory regulators (Ghisletti et al., 2007; Saijo et al., 2013) while others have shown that PPAR-γ can regulate the secretion of inflammatory cytokines in microglia, ultimately promoting tissue repair (Chawla, 2010; Chinetti-Gbaguidi et al., 2011).

In the present study, we found that adiponectin may contribute to the function and polarization of microglia through PPAR-γ signaling. In particular, adiponectin may play a pro- inflammatory role through PPAR-γ by mediating AdipoR1. Even though we did not present evidence on memory improvement by adiponectin through PPAR-γ in AD brain, we speculate that adiponectin may improve memory in AD by attenuating PPAR-γ signaling in microglia, and regulate the function of microglia under Aβ toxicity by regulating PPAR-γ through AdipoR1.

Taken together, we conclude that: (1) adiponectin may induce the M2 polarization of microglia in Aβ toxicity; (2) adiponectin may regulate the production of pro-inflammatory cytokines by mediating AdipoR1 under Aβ toxicity; and (3) adiponectin may exert anti-inflammatory functions in microglia and regulate the polarization of microglia through PPAR-γ signaling under Aβ toxicity. Thus, we suggest that adiponectin is a crucial adipokine to attenuate AD pathology by regulating the function of microglia.

## Author Contributions

JS conducted the experiments and contributed to the writing of the preliminary draft. S-MC conducted the experiments. BCK designed the study and wrote the manuscript, and provided overall supervision for the project.

## Conflict of Interest Statement

The authors declare that the research was conducted in the absence of any commercial or financial relationships that could be construed as a potential conflict of interest.
